# Handbook of Comparative Histology and Physiology of the Domestic Animals

**Published:** 1884-10

**Authors:** 


HANDBOOK OF COMPARATIVE HISTOLOGY AND PHYSIOLOGY OF THE DOMESTIC ANIMALS. Volume I. HISTOLOGY OF THE DOMESTIC ANIMALS for veterinarians and students. Edited by Dr. W. Ellenberger, Professor in the Royal Veterinary School of Dresden. Assisted by Professors Bonnet, of Munich, Czokor, of Vienna, Eichbaum, of Giessen, Eversbusch, of Munich, Flesch, of Berne, Kitt, of Munich, Schutz, of Berlin, Sussdorf, of Stuttgart, and Docent Perez, of Hanover. Part I. With 204 illustrations. Berlin: Paul Parey. 1884. 8vo, pp. 308. Price M. 12, about $3.00.

We greet with great pleasure the announcement of the publication of the present work. It covers a subject that has not yet been worked up in the systematic manner proposed by Professor Ellenberger, and it will fill a want very much felt. There are a good many German veterinarians in this country to whom the book in the original will be all that is needed. For the rest we sincerely trust that some English or American publisher will find it worth while to undertake a translation.

The editor proposes in four compact volumes to cover the subject of the microscopic anatomy and the physiology of the domestic animals. He has secured as helpers a number of eminent gentlemen, many of whose names are familiar on this side of the Atlantic.

The first part, now before us, treats of the histology of the cell, of the tissues in general, of general organology, and of the renal and genital apparatus. There is little to criticise and much to admire in this volume. The illustrations are numerous, excellently drawn, and in most cases original.

We commend Prof. Ellenberger's work most heartily to our readers.

